# Mitochondrial-Endothelial Crosstalk in Cardiometabolic Disease: Mechanisms and Translational Opportunities in the Multi-omics Era

**DOI:** 10.31083/RCM47549

**Published:** 2026-05-25

**Authors:** Di Liu, Jiaxi Sun, Yuwei He, Yuhang Sun, Mengjie Yin, Jie Zhang, Xin Zhao

**Affiliations:** ^1^The First Clinical College, Liaoning University of Traditional Chinese Medicine, 110847 Shenyang, Liaoning, China; ^2^Department of Cardiology, The Second Hospital of Dalian Medical University, 116023 Dalian, Liaoning, China

**Keywords:** mitochondria, endothelial dysfunction, cardiometabolic diseases, multi-omics, translational therapy

## Abstract

Mitochondria and endothelial cells engage in bidirectional crosstalk to maintain vascular tone, barrier integrity, and inflammatory quiescence. In cardiometabolic diseases (CMDs), metabolic overload and chronic inflammatory cues disrupt endothelial mitochondrial bioenergetics, dynamics, and quality-control mechanisms. As protective systems weaken, redox imbalance and impaired nitric oxide signaling—further exacerbated by barrier dysfunction—trigger endothelial activation and loss of homeostasis. Clinical translation has lagged largely because endothelial responses vary across vascular beds and microenvironments, and most clinical trials fail to align patient selection or endpoints with mitochondrial mechanisms. This review addresses a major translational gap: how mitochondrial stress programs map onto context-specific endothelial phenotypes in human CMDs, and how this mapping can inform the selection of actionable therapeutic strategies. Indeed, this review integrates single-cell and spatial multi-omics data to link mitochondrial stress and metabolic remodeling to specific anatomical niches, transforming the broad notion of “endothelial dysfunction” into defined biological programs for biomarker selection and target discovery. Moreover, this review categorizes translational opportunities by the strength of human evidence. Near-term priorities include repurposed cardiometabolic drugs (e.g., sodium-glucose cotransporter 2 (SGLT2) inhibitors, glucagon-like peptide-1(GLP-1) receptor agonists) and circulating biomarkers for patient stratification or pharmacodynamic monitoring (e.g., growth differentiation factor 15 (GDF15), cell-free mitochondrial DNA (cf-mtDNA), endothelium-derived extracellular vesicles). In contrast, gene and cell therapies, as well as advanced delivery and regenerative platforms, remain at the preclinical stage and require stronger mechanistic validation, improved safety profiles, and scalable delivery systems before clinical evaluation. Thus, a key unmet need is for multicenter, mechanism-informed trials that integrate endothelial functional endpoints (e.g., flow-mediated dilation (FMD)/peripheral arterial tonometry (PAT) with mitochondrial-associated molecular readouts under harmonized protocols and standardized reference criteria to enhance reproducibility and cross-study comparability. Collectively, these insights establish mitochondrial–endothelial biology as an evidence-based entry point for precision vascular medicine in CMDs.

## 1. Introduction

Cardiometabolic diseases (CMDs)—including dyslipidemia, hyperglycemia, obesity, and hypertension—remain a major global health burden [1]. Although age-standardized cardiovascular mortality has declined, Global Burden of Disease (GBD) projections indicate that absolute cardiovascular deaths will continue to rise through 2050, largely driven by population growth and aging [2, 3, 4]. These trends underscore the need to better define early vascular alterations in CMDs and to identify targets that can support prevention and more precise clinical management.

Endothelial dysfunction (ED) is a common early abnormality in CMDs and is linked to vascular complications such as atherosclerosis [5, 6]. Despite the relatively low mitochondrial volume fraction in endothelial cells (ECs), mitochondrial function is increasingly recognized as relevant to endothelial homeostasis and adaptation to metabolic stress [7, 8, 9]. Across experimental and clinical studies, alterations consistent with disturbed mitochondrial homeostasis are frequently reported alongside ED in diverse cardiometabolic settings. However, the extent to which these observations reflect shared principles across vascular beds and disease contexts remains unclear.

This uncertainty is reinforced by an emphasis on individual pathways, which has limited our ability to account for endothelial heterogeneity, tissue specificity, and microenvironmental influences in CMDs [10]. Recent advances in single-cell and spatial transcriptomics, together with proteomic and metabolomic profiling, now enable higher-resolution characterization of endothelial diversity and metabolic features *in vivo* [11, 12]. Nevertheless, clinical translation of therapies targeting mitochondrial metabolism has been constrained by small cohorts and marked phenotypic heterogeneity. More importantly, studies rarely incorporate systematic multi-omics stratification, making it difficult to link candidate biomarkers to underlying mechanisms and to identify likely responders. This review synthesizes the evidence linking mitochondrial homeostasis to ED in CMDs. By identifying critical knowledge gaps to motivate future hypotheses, we systematically evaluate emerging biomarkers and therapies according to their clinical development stage to prioritize directions for precision medicine.

## 2. Vascular Endothelial Dysfunction

ED constitutes the functional and pathological foundation of CMDs. Beyond impaired vasodilation, ED encompasses a coordinated disruption of vasomotor balance, barrier integrity, inflammatory activation, and maladaptive phenotypic transitions [5, 6].

In CMDs, one of the earliest and most consequential abnormalities is a redox-driven collapse of nitric oxide (NO) signaling, which shifts the vasomotor set point from dilation to constriction [13]. Excess oxidant production—arising from NADPH oxidases and mitochondrial electron leak—rapidly quenches NO and promotes peroxynitrite formation, which in turn oxidizes tetrahydrobiopterin (BH4) and drives endothelial nitric oxide synthase (eNOS) uncoupling [14, 15]. Once uncoupled, eNOS shifts from an NO-generating enzyme toward a net source of reactive species, amplifying endothelial oxidative injury and further reducing NO bioavailability [16, 17, 18, 19]. In parallel, endothelin-1 (ET-1) signaling sustains vasoconstrictor tone and progressively erodes vasodilator reserve [20, 21]. Although endogenous antioxidant programs are often insufficient to offset persistent metabolic dysregulation [22, 23, 24]. Clinically, the NO/redox axis correlates directly with impaired endothelium-dependent vasodilation and can be monitored via functional measures such as flow-mediated dilation (FMD). Restoring NO bioavailability at this early stage represents a tractable therapeutic target before inflammatory and structural changes become irreversible [25, 26].

Endothelial injury in CMDs involves barrier dysfunction, marked by glycocalyx shedding, junctional disruption, and inflammatory activation, triggered by metabolic and inflammatory stress [27, 28]. Loss of the luminal glycocalyx disrupts mechanochemical crosstalk and attenuates laminar shear-stress sensing, leading to suppression of the vasoprotective transcription factors Krüppel-like factor 2 (KLF2) and Krüppel-like factor 4 (KLF4) [29]. This repression extends to peroxisome proliferator-activated receptor gamma coactivator 1-alpha (PGC-1α), impairing mitochondrial biogenesis and reducing endothelial bioenergetic capacity via defective mechanotransduction [29]. Barrier deterioration is further driven by Yes-associated protein/transcriptional coactivator with PDZ-binding motif (YAP/TAZ) activation and vascular endothelial cadherin (VE-cadherin) destabilization, which induce cytoskeletal remodeling and bias mitochondria toward a fragmented, fission-dominant state [30]. Notably, this YAP/TAZ-to-mitochondrial remodeling pathway represents a cross-scale mechanobiological pathway in CMDs. In this pathway, tissue-level mechanical cues are transduced into organelle-level bioenergetic and inflammatory phenotypes. This is an emerging frontier with direct therapeutic relevance. A key consequence is the loss of tonic restraint on inflammatory programs: nuclear factor-kappa B (NF-κB) signaling becomes permissive, whereas mitochondrial fragmentation increases reactive oxygen species (ROS) production and exacerbates barrier damage in a feed-forward loop [31, 32, 33]. Consistent with this shift, adhesion molecules such as intercellular adhesion molecule-1 (ICAM-1) and vascular cell adhesion molecule-1 (VCAM-1) are upregulated and displayed on the endothelial surface, transforming the endothelium from a quiescent interface into an active recruiter of leukocytes [34]. Circulating markers such as syndecan-1 and soluble VCAM-1/ICAM-1 correlate with disease severity and may serve as accessible biomarkers of endothelial barrier dysfunction [35, 36]. Thus, barrier injury is not merely an epiphenomenon of ED; it serves as a mechanistic link between impaired mechanosensing, mitochondrial stress, and inflammation in CMDs.

Under prolonged stress, ED can become persistent, characterized by phenotypic plasticity, regulated cell death, and microvascular rarefaction. Endothelial-to-mesenchymal transition (EndMT), marked by loss of endothelial markers like cluster of differentiation 31 (CD31) and gain of mesenchymal traits such as alpha-smooth muscle actin (α-SMA), reflects a cellular reprogramming that links extracellular matrix remodeling to altered mitochondrial dynamics [37, 38]. Fibrotic stiffening may limit mitochondrial fusion, while the mesenchymal program elevates anabolic demand, driving cells away from quiescence toward a biosynthetically active metabolic state. Regulated cell death pathways, particularly pyroptosis and ferroptosis, propagate vascular inflammation through the release of damage-associated molecular patterns (DAMPs) [39, 40]. As endothelial loss accumulates due to impaired angiogenesis (e.g., defective vascular endothelial growth factor (VEGF) signaling) and reduced endothelial progenitor cells, microvascular rarefaction often develops [41, 42]. Capillary loss establishes chronic tissue hypoxia, suppressing mitochondrial biogenesis and enforcing a glycolytic shift that locks the endothelium into a state of metabolic inflexibility. The emergence of EndMT and rarefaction marks a therapeutic turning point: ED transitions from reversible functional changes to irreversible structural damage. This shift implies the insufficiency of vasodilator-centric interventions alone, emphasizing the necessity for integrated therapeutic approaches that concurrently mitigate inflammatory cell death, maintain microvascular integrity, and restore mitochondrial function.

## 3. Functional Role of Mitochondria in Endothelial Cell Physiology and Pathology

**Fig. 1.**
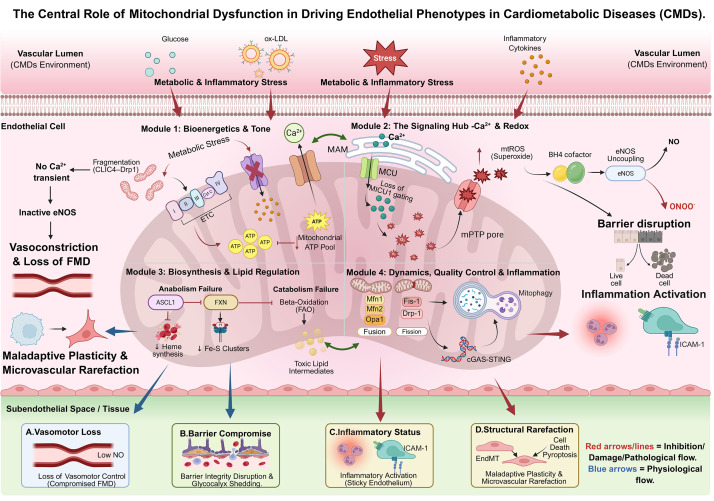
**The central role of mitochondrial dysfunction in driving endothelial phenotypes**. The schematic maps four mitochondrial functional defects to distinct endothelial pathologies. (A) Bioenergetic failure (low ATP) impairs calcium signaling, leading to vasoconstriction. (B) Dysregulated Ca^2+^ buffering and mtROS surges compromise the glycocalyx and barrier integrity. (C) Collapse of quality control allows mtDNA leakage to activate the cGAS–STING inflammatory axis. (D) Metabolic remodeling and biosynthetic failure drive endothelial-to-mesenchymal transition (EndMT) and capillary loss. CMDs, cardiometabolic diseases; ox-LDL, oxidized low-density lipoprotein; CLIC4, chloride intracellular channel 4; Drp1, dynamin-related protein 1; Ca^2+^, calciumion; MAM, mitochondria-associated membrane; MCU, mitochondrial calcium uniporter; MICU1, mitochondrial calcium uptake 1; mtROS, mitochondrial reactive oxygen species; BH4, tetrahydrobiopterin; eNOS, endothelial nitric oxide synthase; NO, nitric oxide; ONOO⁻, peroxynitrite; mPTP, mitochondrial permeability transition pore; ETC, electron transport chain; ATP, adenosine triphosphate; ASCL1, achaete-scute family bHLH transcription factor 1; FXN, frataxin; Fe-S, iron-sulfur; FAO, fatty acid β-oxidation; Mfn1, mitofusin 1; Mfn2, mitofusin 2; Opa1, optic atrophy 1; Fis-1, mitochondrial fission 1 protein; cGAS–STING, cyclic GMP-AMP synthase–stimulator of interferon genes; ICAM-1, intercellular adhesion molecule-1; FMD, flow-mediated dilation. Fig. 1 was created with BioRender.com.

Although mitochondria occupy only ~5% of endothelial cytoplasmic volume, their dominant role in the endothelium extends beyond bulk ATP provision to environmental sensing and signal integration. In CMDs, impaired mitochondrial bioenergetics, Ca^2+^-redox coupling, metabolism, and quality control lead to a common endothelial phenotype: dysfunctional vasoregulation, barrier fragility, and inflammation. Instead of listing numerous intermediate pathways and druggable nodes, we focus on the key mechanistic bottlenecks that best explain these phenotypic abnormalities (examples in Fig. 1).

Endothelial mitochondria help sustain spatially restricted ATP microdomains that support rapid vasodilatory signaling. Although endothelial basal metabolism primarily relies on glycolysis, mitochondrial-derived ATP pools sustain ATP-dependent calcium pumps and kinases, which are essential for eNOS activation and nitric oxide production to mediate vasodilation [43, 44]. In line with this compartmentalized model, selective inhibition of mitochondrial ATP synthase disrupts Ca^2+^ transients, reduces nitric oxide output, and impairs vasodilation—even when global ATP remains preserved by glycolysis. Under sustained metabolic stress, mitochondrial fragmentation (e.g., via pathological chloride intracellular channel 4 (CLIC4)–Drp1 interactions) can further destabilize these microdomains and compromise Ca^2+^ handling and NO signaling [45]. Importantly, mitochondrial reliance varies across vascular beds; restoring oxidative phosphorylation support may therefore be particularly relevant in endothelial populations with higher mitochondrial dependence, such as hepatic sinusoidal endothelial cells [11].

Beyond ATP microdomains, a single integrated axis links mitochondrial Ca^2+^ dysregulation to redox imbalance, endothelial barrier failure, and inflammation [46, 47, 48, 49]. In CMDs, impaired mitochondrial Ca^2+^ handling increases ROS, depletes BH4, uncouples eNOS, and reduces NO bioavailability, directly impairing vasodilation [50, 51]. In parallel, oxidative stress destabilizes junctional architecture (e.g., VE-cadherin dependent adherens junctions), increasing permeability and barrier leak [52, 53]. Non-energetic mitochondrial functions—such as heme and iron–sulfur cluster biogenesis and lipid metabolism—contribute to endothelial homeostasis and can worsen dysfunction when disrupted [54, 55, 56]. Finally, defective quality control mechanisms, such as fission-fusion imbalance and impaired mitophagy, cause dysfunctional mitochondria to accumulate and release mitochondrial DNA (mtDNA). This activates the cyclic GMP-AMP synthase–stimulator of interferon genes (cGAS–STING) pathway and inflammasomes, turning local metabolic stress into chronic endothelial inflammation and immune activation [57, 58, 59, 60]. These mechanisms suggest that restoring endothelial function in CMDs requires not only correcting downstream signaling but also targeting upstream mitochondrial metabolism, biosynthesis, and quality control in a vascular bed–specific way.

## 4. Multi-omics Approaches in the Study of Mitochondrial Interactions in ECs

**Fig. 2.**
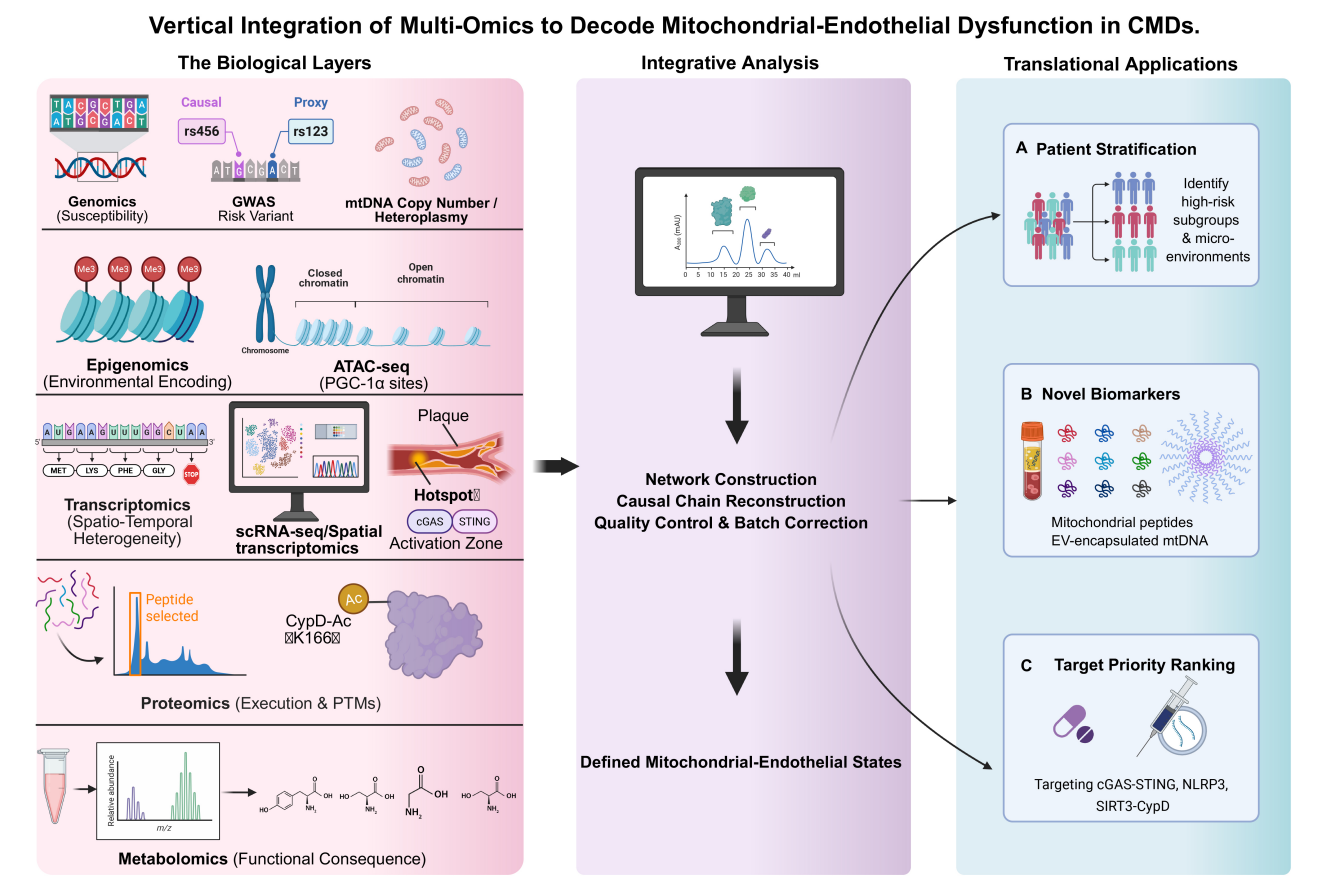
**An ideal multi-omics pipeline for endothelial–mitochondrial biology**. The pipeline illustrates a six-step strategy for vertical integration: (1) Establishment of deeply phenotyped cohorts with standardized biospecimen collection; (2) Parallel acquisition of multi-layer data (genomics, epigenomics, transcriptomics, proteomics, metabolomics) from matched samples; (3) Rigorous quality control and harmonization with clinical hemodynamic variables; (4) Computational network integration to derive latent ‘Mitochondrial-Endothelial States’; (5) Construction of composite biomarker scores; and (6) Validation through perturbation experiments in model systems and replication in independent clinical cohorts. GWAS, genome-wide association studies; mtDNA, mitochondrial DNA; PTMs, post-translational modifications; PGC1α, peroxisome proliferator-activated receptor gamma coactivator 1-alpha; ATAC-seq, assay for transposase-accessible chromatin with sequencing; scRNA-seq, single-cell RNA sequencing; cGAS–STING, cyclic GMP-AMP synthase–stimulator of interferon genes; PTMs, post-translational modifications; CypD-Ac, acetylated cyclophilin D; K166, lysine 166; EV, extracellular vesicle; NLRP3, NOD-like receptor family pyrin domain-containing 3; SIRT3, sirtuin 3; CypD, cyclophilin D. Fig. 2 was created with BioRender.com.

The mitochondrial mechanisms outlined above unfold within highly heterogeneous endothelial states *in vivo*. In CMDs, ED is not a uniform condition but a spectrum shaped by vascular bed identity and the surrounding microenvironment. This heterogeneity also limits single-layer omics: transcriptional stress signatures do not necessarily predict protein activity or metabolite availability. The main value of multi-omics, therefore, is not technical cataloging per se, but the ability to define endothelial “mitochondrial states” at single-cell resolution, map their tissue context, and prioritize cross-layer biomarkers and targets with clinical operational value (see Fig. 2 for an overview).

Genomic signals such as mtDNA copy number variation and heteroplasmy are associated with ED risk at the susceptibility level [61]. Such predisposition can be reinforced through nuclear-mitochondrial retrograde signaling and epigenetic remodeling (e.g., DNA methylation) [62, 63]. Under oxidative and metabolic stress, persistent changes in chromatin accessibility may encode “stress memory” [64, 65]. These dynamic epigenomic features may support risk stratification and provide sensitive monitoring readouts for interventions intended to “reset” maladaptive metabolic programs [66].

Spatial and single-cell profiling further reveals that mitochondrial stress is not evenly distributed: it concentrates in distinct endothelial subpopulations and in high-risk microanatomic regions (e.g., plaque shoulders) that drive lesion progression [67, 68, 69]. In such niches, mtDNA release and cGAS–STING activation can co-localize with inflammatory markers (e.g., VCAM-1), illustrating *in situ* coupling between metabolic stress and innate immune activation [70]. Because transcription alone is insufficient to infer function, proteomics and post-translational modification (PTM) profiling are essential to validate the effector layer; for example, SIRT3/GCN5L1-driven acetylation of cyclophilin D acts as a switch for mitochondrial permeability transition pore (mPTP) opening [71, 72]. These multi-dimensional evidences refine “endothelial dysfunction” from a broad label to a detailed mitochondrial-inflammation phenotype map that can be sampled and targeted. Finally, metabolomics provides the closest real-time functional readout. The depletion of the nicotinamide adenine dinucleotide (NAD^+^) pool and the reduction of glutathione (GSH) often precede obvious structural damage and are highly potential early warning indicators [73, 74]. Multi-omics integrates inherited susceptibility, spatially resolved cell states, effector capacity, and metabolite availability, refining ED from a broad label into actionable mitochondrial–inflammation phenotypes and clinically tractable biomarkers [75].

## 5. Translational Prospects

**Fig. 3.**
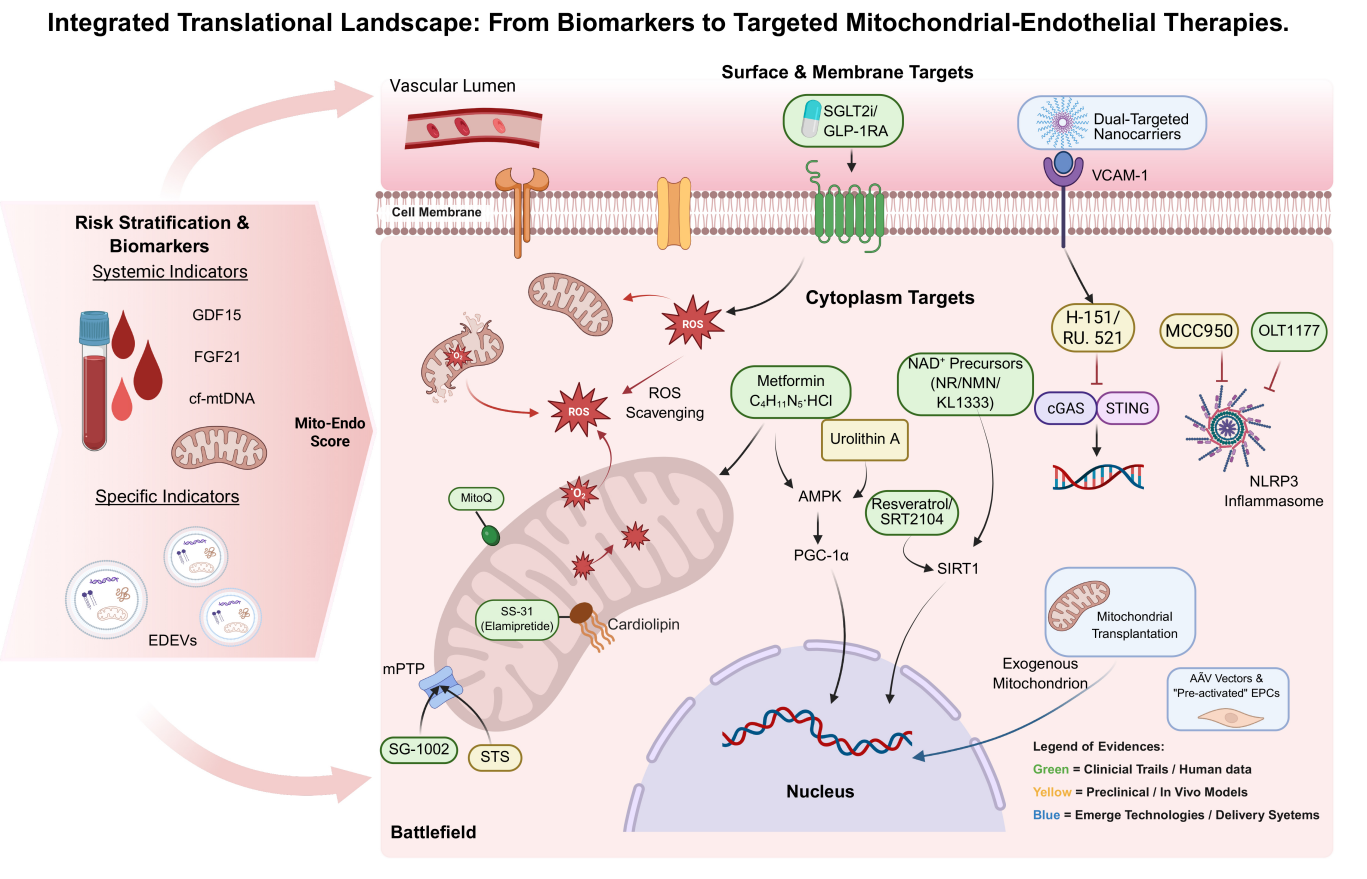
**Schematic of biomarkers and targeted therapies**. The figure illustrates the progression from risk stratification to subcellular targeting. (Left) The “Mito–Endo Score” integrates systemic stress markers and vascular-specific “liquid biopsy” readouts to stratify risk. (Center) Therapeutic strategies are mapped to surface, cytoplasmic, or mitochondrial targets within a stressed endothelial cell. (Legend) Colors indicate evidence tier: Green (Tier 1–2: clinical outcome evidence or early human mechanistic signals), Orange (Tier 3: preclinical candidates requiring human target engagement/validation), and Blue (Tier 4: enabling/emerging technologies with feasibility and safety barriers as the primary gating factors). STS, sodium thiosulfate; EDEVs, endothelial-derived extracellular vesicles; FGF21, fibroblast growth factor 21; EPCs, endothelial progenitor cells; AMPK, AMP-activated protein kinase; SGLT2i, sodium-glucose cotransporter 2 inhibitors; GLP-1RA, glucagon-like peptide-1 receptor agonists; VCAM-1, vascular cell adhesion molecule-1; ROS, reactive oxygen species; NAD^+^, nicotinamide adenine dinucleotide; NR, nicotinamide riboside; NMN, nicotinamide mononucleotide; cGAS, cyclic GMP-AMP synthase; STING, stimulator of interferon genes; NLRP3, NOD-like receptor family pyrin domain-containing 3; AMPK, AMP-activated protein kinase; PGC-1α, peroxisome proliferator-activated receptor gamma coactivator 1-alpha; SIRT1, sirtuin 1; mPTP, mitochondrial permeability transition pore; AAV, adeno-associated virus. Fig. 3 was created with BioRender.com.

While the mitochondrial–endothelial axis is strongly supported mechanistically, translational credibility depends on ranking by the strength of human evidence [76]. We prioritize therapeutic opportunities in four tiers based on human evidence strength (Table 1, Ref. [77, 78, 79, 80, 81, 82, 83, 84, 85, 86, 87, 88, 89, 90, 91, 92, 93, 94, 95, 96, 97, 98, 99, 100]), with Fig. 3 providing the comprehensive drug network. Tier 1 and Tier 2 target interventions with proven cardiovascular outcomes or early mechanistic signals, while Tier 3 and Tier 4 cover preclinical candidates and exploratory delivery platforms needing validation. This classification anchors translation to measurable mitochondrial-endothelial endpoints, moving beyond speculative claims toward testable clinical hypotheses.

**Table 1.** **Representative therapeutic strategies targeting the mitochondrial-endothelial axis**.

Strategy category	Representative agents	Target mechanism	Evidence level
Repurposed cardiometabolic drugs	SGLT2i [77, 78], GLP-1RA [79, 80], Metformin [81, 82, 83, 84]	Restore mitophagy (AMPK/ULK1); ↓ mtROS and inflammation	Tier 1: Large clinical outcome trials
Mito-targeted cytoprotectants	SS-31 (Elamipretide) [85], MitoQ [97]	Stabilize cardiolipin; ↓ mtROS scavenging	Tier 2: Early mechanistic human evidence (Phase I–II; small RCTs)
Metabolic and redox modulators	NAD^+^ Precursors [88] (NR/NMN [86, 87]), H_2_S Donors (SG-1002) [98]	↑ SIRT1 activity; ↑ eNOS coupling	Tier 2: Early mechanistic human evidence (Phase I–II; small RCTs)
Innate immune and mitophagy modulators	Innate immune gating (e.g., NLRP3 [99]/cGAS–STING inhibitors) [91, 92];	↓ Inflammasome activation;	Tier 3: Preclinical; requires human target engagement
	Mitophagy enhancers (e.g., urolithin A) [89, 90]	↑ Mitophagy (UPRmt)	
Advanced gene and cell platforms	Endotheliotropic AAVs [100], EPC/ECFC Therapy [96]	Vascular-specific gene delivery; Microvascular restoration	Tier 3–4: Predominantly Tier 4 enabling platforms; preclinical/early feasibility with substantial delivery and safety barriers
Direct repair technologies	Mitochondrial Transplantation [93], Nanocarriers [94, 95]	Direct organelle transfer; Precise mitochondrial delivery	Tier 4: Emerging platforms; feasibility and safety first

Representative agents are summarized here, with a complete mechanistic mapping provided in Fig. 3. ECFCs, endothelial colony-forming cell; SIRT1, sirtuin 1; ULK1, Unc-51-like kinase1; RCT, randomized controlled trial; NR/NMN, nicotinamide riboside/nicotinamide mononucleotide; NLRP, NOD-like receptor family pyrin domain-containing; AAV, adeno-associated virus.

Clinically, this requires moving beyond systemic stress readouts (e.g., growth differentiation factor 15 (GDF15) or cell-free mitochondrial DNA (cf-mtDNA)) toward more vascular-specific “liquid biopsy” signals such as endothelium-derived extracellular vesicles (EDEVs), which better capture real-time endothelial injury [101, 102, 103, 104, 105]. A pragmatic near-term approach is the combinatorial use of cf-mtDNA and EDEVs to calibrate systemic metabolic burden against ongoing vascular injury, validated in human induced pluripotent stem cell (iPSC)-derived vascular organoids [106, 107, 108].

The most immediate focus (Tier 1) is the repurposing of sodium-glucose cotransporter 2 (SGLT2) inhibitors [77, 78], GLP-1 receptor agonists [79, 80] and Metformin [81, 82, 83, 84] supported by cardiovascular outcome data and consistent mechanistic links to reduced mtROS and improved mitophagy. For Tier 2 candidates with early human mechanistic signals (e.g., SS-31 or NAD^+^ augmentation), the next step should be small, mechanism-anchored trials that quantify target engagement and define the therapeutic window for reversible dysfunction [85, 86, 87, 88].

Tier 3 approaches should be gated by clear human target engagement and safety before escalation (e.g., pathway-specific innate immune or mitophagy-modulating strategies), with representative candidates summarized in Table 1 [89, 90, 91, 92]. Finally, Tier 4 technologies aim to directly repair compromised endothelial networks via targeted delivery platforms (e.g., dual-targeted nanocarriers or endotheliotropic viral vectors) [93, 94, 95, 96]. The main barriers to clinical advancement remain endothelial targeting specificity, manufacturability, durability of effect, and long-term immunogenicity. A biomarker-defined enrichment strategy coupled to explicit mitochondrial target-engagement endpoints provides the most coherent route to trial design that links mechanistic rescue to clinically interpretable vascular benefit.

## 6. Conclusion

Mitochondrial and ED in CMDs form a coupled, bidirectional system that amplifies vascular injury. Metabolic stress impairs endothelial mitochondrial bioenergetics and quality control, increases oxidative stress, and reduces nitric oxide bioavailability, thereby weakening barrier integrity and microvascular perfusion. As microvascular capacity declines, malperfusion and hypoxia further suppress mitochondrial function and reinforce cardiometabolic dysregulation, ultimately contributing to microvascular rarefaction. The key translational challenge is to define when dysfunction remains reversible in which this trajectory remains reversible versus the transition point at which it progresses to fixed remodeling that becomes less responsive to vasodilator-based strategies.

Recent single-cell, spatial, and multi-omics studies can replace the monolithic concept of “ED” with discrete, interpretable endothelial mitochondrial programs, enabling biomarker prioritization and mechanism-based responder enrichment. However, human evidence still trails mechanistic plausibility, making standardization the immediate next step. Mechanism-driven randomized trials are crucial for assessing well-defined CMDs phenotypes. They should include prespecified functional endpoints and mitochondrial biomarkers like cf-mtDNA, with all procedures following standardized protocols and reproducibility standards. Together, this approach can move the field from association toward phenotype-specific strategies for durable vascular protection.

## Abbreviations

CMDs, cardiometabolic diseases; GBD, Global Burden of Disease; ECs, endothelial cells; ED, endothelial dysfunction; NO, nitric oxide; eNOS, endothelial nitric oxide synthase; ROS, reactive oxygen species; mtROS, mitochondrial reactive oxygen species; EV, extracellular vesicle; NAD^+^, nicotinamide adenine dinucleotide; FMD, flow-mediated dilation; PGI_2_, prostaglandin; ET1, endothelin-1; TXA_2_, thromboxane A_2_; BH4, tetrahydrobiopterin; VSMC, vascular smooth muscle cells; sGC, soluble guanylate cyclase; PKG, protein kinase G; O2⋅⁻, superoxide; ONOO^–^, peroxynitrite; NOX, NADPH oxidase; SIRT1, sirtuin 1; ox-LDL, oxidized low-density lipoprotein; CAD, coronary artery disease; ACS, acute coronary syndrome; NF-κB, nuclear factor-kappa B; DAMPs, damage-associated molecular patterns; eGC, endothelial glycocalyx; EndMT, endothelial-to-mesenchymal transition; GSH, glutathione; ECFCs, endothelial colony-forming cells; MCU, mitochondrial calcium uniporter; TCA, tricarboxylic acid; ΔΨm, mitochondrial membrane potential; FAO, fatty acid β-oxidation; GWAS, genome-wide association studies; WGS, whole genome sequencing; mtDNA, mitochondrial DNA; scRNA-seq, single-cell transcriptomics; ST, spatial transcriptomics; PTMs, post-translational modifications; mPTP, mitochondrial permeability transition pore; MDPs, mitochondria-derived peptides; EDEVs, endothelial-derived extracellular vesicles; H_2_S, hydrogen sulfide; STS, sodium thiosulfate.
